# Is It Possible to Determine Plasma Cytomegalovirus DNA Cut-off and Develop a Scoring System to Predict Cytomegalovirus Gastrointestinal Disease?

**DOI:** 10.5152/tjg.2025.24507

**Published:** 2025-10-06

**Authors:** Gizem Karahan, Sehnaz Alp, Merve Kasikci, Alpaslan Alp, Cenk Sokmensuer, Taylan Kav

**Affiliations:** 1 Department of Infectious Diseases and Clinical Microbiology, Hacettepe University Faculty of Medicine, Ankara, Türkiye; 2 Department of Biostatistics, Hacettepe University Faculty of Medicine, Ankara, Türkiye; 3 Department of Medical Microbiology, Hacettepe University Faculty of Medicine, Ankara, Türkiye; 4 Department of Pathology, Hacettepe University Faculty of Medicine, Ankara, Türkiye; 5 Department of Gastroenterology, Hacettepe University Faculty of Medicine, Ankara, Türkiye

**Keywords:** Cytomegalovirus, gastrointestinal disease, polymerase chain reaction, scoring system

## Abstract

**Background/Aims:**

Early diagnosis of cytomegalovirus gastrointestinal disease (CMV-GID) is often deemed critical for the appropriate management of the disease. Although histopathological examination is the gold standard for diagnosis, non-invasive and early diagnostic methods may be needed to predict CMV-GID. As a rapid and non-invasive method, the detection of CMV DNA in plasma by polymerase chain reaction (PCR) can be utilized. In this study, the aim was to determine the optimal plasma CMV DNA cut-off value and to develop a scoring system as an adjunct diagnostic method for predicting CMV-GID.

**Materials and Methods:**

In this methodological study, the records of patients who underwent gastrointestinal biopsy and plasma CMV PCR testing were retrospectively reviewed. A scoring system based on multivariate analysis was established as a predictive model for CMV-GID. Receiver operating characteristic analysis was performed to determine the optimal plasma CMV DNA cut-off using the Youden Index and to evaluate the performance measures related to the proposed methods for predicting CMV-GID.

**Results:**

A total of 302 patients (125 with the diagnosis of CMV-GID and 177 without CMV-GID, based on their endoscopic biopsy reports and plasma CMV PCR results) were included. The optimal CMV DNA cut-off value in plasma to predict CMV-GID was determined to be 272 copies/mL. A total score of 28.1 obtained from the predictive model was set to be the optimal value for predicting CMV-GID.

**Conclusion:**

The use of a scoring system and an optimal CMV DNA cut-off may help to predict CMV-GID and facilitate early diagnosis and treatment.

**Main Points**

- Plasma cytomegalovirus DNA levels may guide the diagnosis of cytomegalovirus gastrointestinal disease. In this study, the optimal plasma cytomegalovirus DNA cut-off value was found to be 272 copies/mL for predicting cytomegalovirus gastrointestinal disease.
- A scoring system was proposed to predict cytomegalovirus gastrointestinal disease. Accordingly, the optimal cut-off value to predict cytomegalovirus gastrointestinal disease was determined to be >28 points out of 56 points.
- Each center should determine its optimal cut-off value with the help of the adopted test methodology to guide the diagnosis and treatment of its patient population.
- Real-life experiences and multicenter large-scale prospective research are needed to confirm the accuracy of both the cut-off values and the scoring system proposed in this study.

## Introduction

Cytomegalovirus (CMV) infection, especially in an immunosuppressed host, can be severe and may progress to a fatal clinical course with tissue and organ involvement. Cytomegalovirus gastrointestinal disease (CMV-GID) can worsen the prognosis of some diseases and increase the length of hospital stay, need for colectomy, and mortality in patients, particularly in those with inflammatory bowel disease. In addition, perforation, toxic megacolon, and massive bleeding are common complications of CMV-GID.^1^ To prevent these complications and poor outcomes, early diagnosis and rational use of polymerase chain reaction (PCR) testing in disease management appear to be important. Histopathological confirmation of CMV in biopsy specimens is considered the gold standard for the diagnosis of CMV-GID.^2^ Since the definitive diagnosis of the disease relies on histopathological examination, gastrointestinal endoscopy is highly recommended to obtain tissue for analysis.^3^ Detection of CMV DNA in plasma or serum by PCR is proposed as a non-invasive, easy-to-apply adjunct diagnostic tool for predicting CMV disease, with the advantage of providing rapid results. Relevant research often recommends that further studies focus on improving the diagnostic performance for CMV-GID, as identifying serum CMV DNA alone in patients with gastrointestinal complaints yields low sensitivity.^4^ Besides, its diagnostic sensitivity and specificity are reported to vary according to the cut-off values used for CMV DNA levels. In general practice, each center sets a cut-off value for the assay to assist in the decision to initiate antiviral therapy.^5^ Despite a consensus on the typical histopathological or immunohistochemical findings of CMV disease in tissue for definitive diagnosis, improved non-invasive early detection methods to predict CMV-GID for guiding antiviral treatment are still needed.^6^ Therefore, the aim was to develop a scoring system and calculate an optimal plasma CMV DNA cut-off for predicting CMV-GID.

## Materials and Methods

In this methodological study, the database was retrospectively evaluated to identify adult patients (age ≥18 years) with at least one gastrointestinal biopsy report indicating the presence or absence of CMV, along with concomitant plasma CMV PCR testing conducted within 45 days of endoscopic biopsy sampling. This study protocol was approved by the Institutional Non-Interventional Clinical Research Ethics Committee of Hacettepe University (Registration Number: GO 20/868) on November 3, 2020. A database search of patient records dating back to Jan 1, 2007, was done with the help of the information technology department. Since the records of patients were retrospectively reviewed and no prospective follow-up or intervention was performed, written informed consent was not obtained. The setting was a 1000-bed tertiary care university hospital. Individual patient files and medical records were searched, and the following data were obtained and anonymized: age; gender; underlying disorder(s); administered medications; plasma CMV DNA levels; type of gastrointestinal complaints; endoscopic examination findings; histopathological, immunohistochemical staining, and viral and molecular detection test results; laboratory assessment findings; date of gastrointestinal biopsy and plasma CMV PCR sampling. The variables of interest considered in this study are listed in the Supplement File (Annex-1). CMV-GID was defined as the presence of CMV in gastrointestinal tissue specimens documented using histopathology, virus isolation, immunohistochemistry, or DNA hybridization techniques.^7^ Only the first episode of CMV-GID was included if the patient had subsequent episodes. Patients who underwent endoscopic examination while receiving antiviral medication and patients with plasma CMV DNA levels below the detection limit of the test (reported as negative without any numerical value) were excluded. Patients with a histopathological diagnosis of CMV-GID but negative plasma CMV DNA were not excluded so as to avoid bias in the assessment of the diagnostic value of CMV DNA in CMV-GID. Analyses were performed between 2 groups—those with CMV-GID and those without—to determine the cut-off values of CMV DNA levels predicting the presence of CMV-GID. All patients were categorized according to their underlying disease, which constituted the primary risk for the development of CMV-GID. Diagnostic groups were categorised from 1 to 8. The study patients were categorized into 8 subgroups according to their underlying conditions as stated in the following numerical codes: 1 = inflammatory bowel disease (IBD); 2 = solid organ transplantation (SOT); 3 = hematopoietic stem cell transplantation (HSCT); 4 = solid organ malignancy; 5 = hematologic malignancy; 6 = Human immune deficiency virus/Acquired immune deficiency syndrome (HIV/AIDS); 7 = rheumatologic and autoimmune diseases and primary immunodeficiencies; and 8 = other than listed above (such as immunocompetent but elderly or critically ill patients). If the patient had no immunosuppression considered to increase CMV-GID risk, they were classified as immunocompetent in the eighth and final category. Since there may be differences in CMV viremia between diagnostic groups, the cut-off values were calculated separately for each group. In addition, all the variables mentioned above were compared between the CMV-GID positive and negative groups to determine the risk factors of the disease. The identified risk factors were included in the model in order to develop a scoring system. The recommendations of the Transparent Reporting of a Multivariable Prediction Model for Individual Prognosis or Diagnosis Statement were followed.^8^

### Statistical Analysis

Statistical analyses were performed using IBM® SPSS® v.25 Software (IBM SPSS Corp.; Armonk, NY, USA). A *P*-value of <.05 was considered statistically significant. No missing data corresponding to each variable of interest was reported. The normality of distribution was assessed by visual (histogram and probability charts) and analytical (Kolmogorov-Smirnov test) methods. Accordingly, while normally distributed variables are shown as means (M) ± SD, non-normally distributed data are expressed as medians (minimum-maximum or interquartile range). Categorical variables were demonstrated as numbers (n) and percentages (%) and patients’ demographics were descriptively presented. The categorical variables were compared between the groups using a series of chi-square tests, and pair-wise comparisons of the continuous variables were performed using the Mann–Whitney* U* test. Subgroup analyses were done by stratifying the patients according to their underlying diseases. Spearman’s correlation analysis was used to determine the relationships between the variables. The variables satisfying the criteria (*P* < .20) in the univariate analysis were included in the multivariate analysis, and the logistic regression model was built using the backward elimination method. The validity of the regression model was tested using the “Regression Modeling Strategies” (rms*)* package in the R program.^9^ Variables with *P* < .10 were automatically included in the regression model, and multivariate analysis revealed the intended diagnostic scoring system. A scoring system was developed based on the coefficients of the regression model. Coefficients were multiplied by 10 and rounded to the nearest integer. The adjusted coefficients for every variable in the regression model were combined into a linear equation to obtain scores. C index was computed using the 1000 bootstrap samples. Receiver operating characteristic (ROC) analysis was conducted using the web-based easyROC (v. 1.3.1) (Hacettepe University; Ankara, Turkey) tool to determine the optimal plasma CMV DNA cut-off value derived from the Youden Index to distinguish patients with and without CMV-GID and to assess its performance measures. A post-hoc power analysis was performed using the Power Analysis and Sample Size Software.

## Results

During the study period, among 4895 patients with pathological examination records of gastrointestinal tissue, 302 patients with endoscopic biopsy reports and plasma CMV PCR results obtained within 45 days of biopsy sampling were identified and included in the study. Among them, 125 patients (41%) were diagnosed with CMV-GID. Demographic data and clinical characteristics of patients are presented in Table 1. The most common underlying disorder was IBD (28.8%), followed by HSCT (14.6%), rheumatological and autoimmune diseases or primary immunodeficiencies (13.3%), solid organ malignancy (12.9%), SOT (7.6%), hematological malignancy (5.6%), and HIV/AIDS (5%). The median duration of hospitalization was 21.5 days. The overall mortality rate was 18.2%. The patients with CMV-GID were significantly older (*P* < .001) and had significantly higher steroid use (*P* = .001) than those without CMV-GID, and the use of more than 2 immunosuppressive drugs was significantly higher (*P* = .015) in patients with CMV-GID as well (Table 1). The groups did not significantly differ in terms of gastrointestinal system complaints, except for dyspepsia, which was found to be significantly higher in the CMV-GID group (*P* = .017) (Table s-1). Endoscopic examination findings of ulcers (*P* < .001), exudating ulcers (*P* < .001), ulcers with irregular margins (*P* < .001), large mucosal defects (*P* < .001), bleeding (*P* = .043), and pancolitis (*P* = .003) were significantly higher in the group with CMV-GID (Table 2). The groups did not significantly differ in terms of the site of gastrointestinal involvement, except for the ileum, which was significantly higher in the CMV-GID-negative group (*P* < .05) (Table s-2). While inclusion bodies were reported in 32% of histopathological examinations of endoscopic biopsy specimens of CMV-GID patients, cytomegaly and/or giant cell formation was reported in 12%. As the immunohistochemical examination could not be performed on the biopsy material of 1 patient due to tissue shrinkage, a histopathological diagnosis of CMV-GID was made in this case with the observation of typical inclusion bodies attributed to CMV. Nevertheless, 96% of the remaining 124 CMV-GID patients had positive immunohistochemical staining. There were no significant differences between the groups in terms of laboratory parameters such as hemogram test results, biochemical profiles, and acute phase reactants (Table s-3).

**Figure 1.**
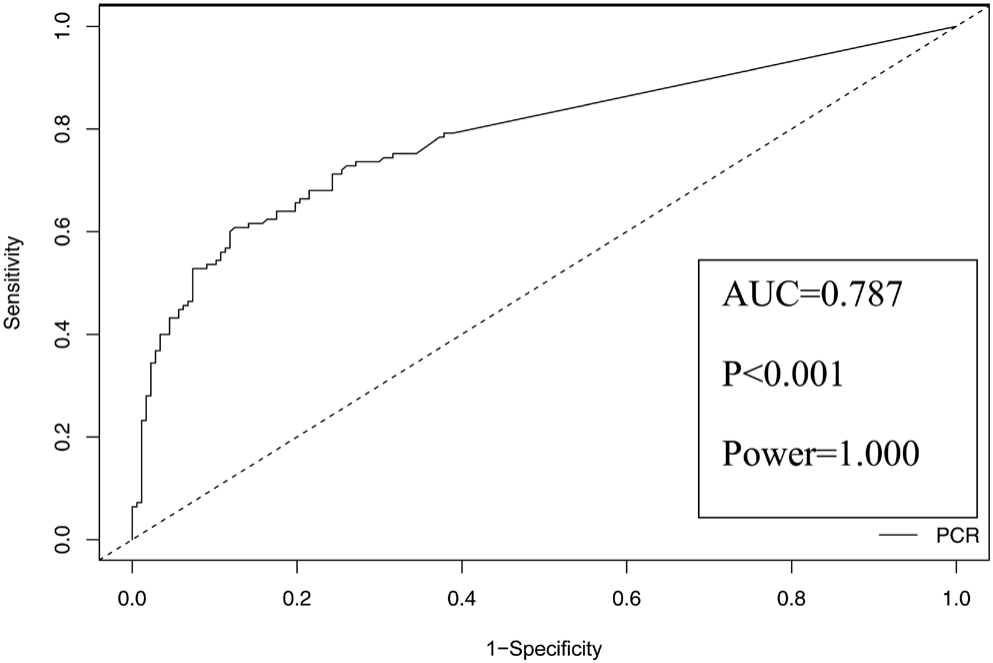
ROC curve of the CMV DNA cut-off value in plasma for the entire cohort.

The median elapsed time between plasma CMV DNA testing and endoscopic biopsy sampling was 5 days. There were 134 patients with positive immunohistochemistry or histopathology but negative plasma CMV DNA. Those without CMV-GID (the patients who don’t have CMV-GID) (n = 108 and 80.6%) and those diagnosed with CMV-GID (n = 26 and 19.4%). Median CMV DNA was 641 cp/mL (0-2352107 cp/mL) in the group diagnosed with CMV-GID and 0 cp/mL (0-58995 cp/mL) in the group without CMV-GID. Based on the Youden Index, the plasma CMV DNA cut-off value for predicting CMV-GID was determined to be 272 copies/mL, with 60.8% sensitivity, 87.6% specificity, 77.6% positive predictive value, and 76.0% negative predictive value. For maximum specificity (92.7%) and sensitivity (79.2%), the threshold values were determined to be 611 copies/mL and 9 copies/mL, respectively (Figure 1) (Table 3).

For each subgroup of patients, the optimal plasma CMV DNA cut-off to predict CMV-GID was defined as 9 copies/mL in IBD (diagnosis code 1); 298 copies/mL in SOT (diagnosis code 2); 290 copies/mL and 875 copies/mL in HSCT (diagnosis code 3); 124 copies/mL in solid organ and hematological malignancies (diagnosis codes 4 and 5); 1447 copies/mL in HIV/AIDS (diagnosis code 6); and 86 copies/mL in rheumatologic, autoimmune disease, and primary immunodeficiency (diagnosis code 7). In the IBD group (n = 87), 9 of 16 patients (56.3%) who used anti-TNF (Tumor necrosis factor) agents were diagnosed with CMV-GID, while 31 of 71 patients (43.7%) who did not use anti-TNF agents were diagnosed with CMV-GID, and the difference was found to be statistically insignificant (*P* = .361).

Considering the subgroup analysis of 16 patients with tissue CMV DNA, a moderate positive correlation between plasma and tissue CMV DNA was found (rho = 0.645; *P* = .007).

**Figure 2.**
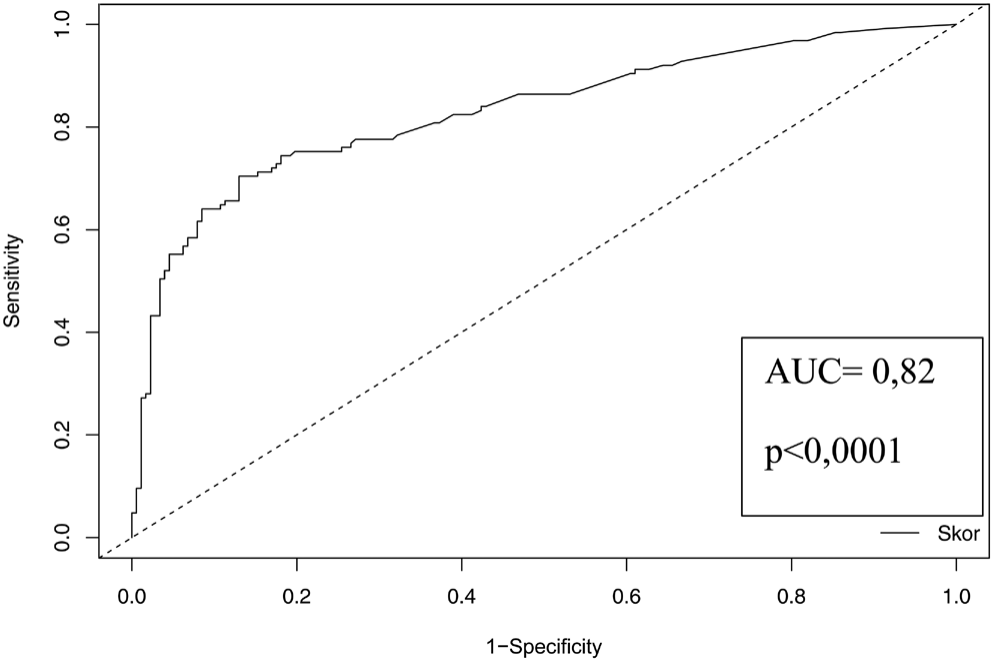
ROC curve of the proposed scoring system to predict the diagnosis of cytomegalovirus gastrointestinal disease.

In the univariate analyses, being older than 65 years contributed to the risk of CMV-GID by 2.22 times; systemic steroid use by 2.36 times; the presence of ulcers, pancolitis, erosion, and granular appearance on endoscopic examination by 2.73 times, 3.34 times, 1.63 times, and 1.85 times, respectively (Table 4). Accordingly, 1 (patient) gets 8 points for being older than 65 years, 5 points for systemic steroid use, 9 points for erosion, 11 points for ulcers, 6 points for pancolitis, 10 points for granular appearance, and 7 points for an increase of 1 log_10_ copies/mL viral load (Table 5). The validity of the regression model through the rms package in the R program and, accordingly, the bootstrapped c-index (number of repetitions = 1000) was found to be 0.825, and the *P*-value was <.0001 (95% CI: 0.776-0.871). The optimal cut-off value for the suggested score to predict CMV-GID was determined to be 28.1 according to the Youden Index. For maximum specificity (96%) and sensitivity (86.4%), the threshold values of the score were determined to be 37.4 and 17, respectively (Figure 2) (Table 6).

## Discussion

The optimal cut-off value for plasma CMV DNA was investigated to predict the diagnosis of CMV-GID, and a scoring system was established as a diagnostic adjunct. Performance measures of the proposed cut-off value and the scoring model were also assessed. The optimal plasma CMV DNA cut-off was set as 272 copies/mL, which could predict CMV-GID with 61% sensitivity and 88% specificity. The proposed scoring system can predict the diagnosis of CMV-GID with 70% sensitivity and 87% specificity.

In the presence of local intestinal inflammation, such as IBD, CMV-GID can develop in the absence of viremia by reactivating the persisting virus in the cells lining the host’s gastrointestinal mucosa.^4^ HIV/AIDS patients, for example, appear to have more severe viremia due to the nature of the disease. The CMV DNA cut-off values appear to be quite different from one group to the other. Therefore, patients in the cohort were divided into diagnostic subgroups, and cut-off values were calculated separately.

Previous studies already offered that the tissue CMV PCR cut-off values could help predict CMV-GID in patients with IBD and HSCT.^6^ Also, CMV disease was shown to be predicted through a serum CMV PCR cut-off value in HIV/AIDS patients, regardless of the localization of organs and system involvement.^6^^,^^10^ In this study, optimal CMV DNA cut-off values were offered to predict CMV-GID for other patient populations as well.

Since the preliminary report of cytomegalic inclusion disease in 1961 in a patient with ulcerative colitis, the literature has witnessed a growing scholarly interest in the relationship between inflammatory bowel disease and CMV.^11^ A previous study with 85 patients with IBD concluded that the qualitative positivity of blood CMV PCR and tissue CMV PCR could be a guide for the decision to initiate antiviral treatment.^12^ CMV-GID in patients with IBD are more likely to be refractory to steroid therapy, which was previously found to be associated with tissue CMV DNA greater than 5500 copies/µg, 250 copies/mg-tissue, and 2.5 log_10_ copies/mg-tissue, respectively.^13^^-^^15^ Therefore, it may be proposed that a high tissue CMV DNA will help predict the refractoriness of the inflamed tissue to steroids and that early initiation of antiviral treatment may improve treatment response. In the case of moderate-to-severe colitis, particularly steroid-refractory disease, it is often recommended to investigate CMV-GID on endoscopic biopsy samples through hematoxylin-eosin staining and, if convenient, immunohistochemical methods or to determine the viral load in the tissue by quantitative PCR.^16^

In the relevant literature, only 1 study attempted to propose a blood CMV DNA cut-off value by quantitative PCR to predict CMV-GID among SOT recipients. In that retrospective study, among 494 kidney transplant recipients, 17 patients with CMV-GID and 20 patients without CMV-GID were included. Accordingly, the study concluded the optimal plasma CMV DNA cut-off to be 4.063 IU/mL, with an AUC value of 0.74, a sensitivity of 76.5%, a specificity of 70%, a positive predictive value of 68%, and a negative predictive value of 78%. The study also reported the most prevalent macroscopic findings in the endoscopic examinations as ulcers accompanied by erythematous mucosa, erosion accompanied by erythematous mucosa, nodular erythematous mucosa, and aphthous ulcer. In addition, the frequencies of upper and lower GI (Gastrointestinal) involvement were reported to be similar.^17^

A previous study reported that CMV reactivation would systematically exacerbate inflammatory reactions in the colon and that serum CMV DNA could help predict CMV colitis in patients with IBD.^18^ With the evaluation of a limited number of patients with available tissue CMV DNA (n = 16), a moderate positive correlation between tissue and plasma CMV DNA was found in this study. This positive correlation may imply that the proposed plasma CMV DNA cut-off values can help predict the viral tissue load and, therefore, the disease involvement.

A meta-analysis of 16 studies, 4 of which provided performance measures for blood CMV DNA, reported a pooled sensitivity of 60% and specificity of 100% among 187 patients with IBD.^19^ In a study histopathologically examining postoperative colectomy materials for CMV-GID, CMV reactivation in the tissue occurred in 17 of 77 patients, but none was detected pre-operatively, suggesting that the performance of non-invasive diagnostic tests should be improved.^20^

In a Germany-based study, the researchers attempted to create a scoring system to guide the diagnosis of CMV-GID among patients with IBD through a logistic regression model. Their model covers disease duration, endoscopic Mayo Score, steroid use and dose, TNF-alpha inhibitor use, and the site of disease involvement.^21^D In a different manner, the present study attempted to propose a model to be used not only for patients with IBD but also for other patient groups in the entire cohort, which may be considered the hallmark of this study.

In another retrospective study with 907 IBD cases, it was recommended to use a 2-step scoring system (Berlin Score and Münster Score) and then request a CMV DNA PCR test if necessary, due to the additional financial burden of routine screening of CMV-GID that has a low incidence among patients with IBD.^22^

A 10-year experience at a tertiary referral center that recruited 21 patients to investigate risk factors for the development of CMV-GID during an acute exacerbation of IBD revealed that the absence of leukocytosis, immunosuppressive therapy, and being older than 30 years were associated with CMV-GID. This experience might have led the researchers to recommend frequent screening for CMV infection in the risk group because of the increased risk of mortality due to CMV-GID.^23^ Similarly, the results obtained here demonstrated advanced age and immunosuppressive therapy, primarily systemic steroid use, to be associated with CMV-GID; therefore, these variables were included in the diagnostic scoring system.

As a retrospective and single-center study, there are inherent limitations. Tissue CMV DNA levels could not be obtained for the entire cohort since the patient management could not be intervened due to the retrospective nature of this study. Moreover, despite an adequate overall sample size, some subgroups had a limited number of patients (i.e., poor analysis power in the immunocompetent group) due to the inability to control factors such as patient characteristics and other confounding factors, and as a single-center study, the generalizability of the findings is limited. While the findings are promising, the absence of prospective validation limits the ability to confirm the scoring system and cut-off values in real-world clinical settings.

On the other hand, focusing on a specific end-organ involvement and relying on histopathological examination with an adequate sample size to reach a post-hoc power of over 99%—except for 1 subgroup—can be considered as the strengths of the study. Furthermore, a CMV DNA PCR cut-off value in plasma was offered to predict the diagnosis of CMV-GID and introduce an appropriate scoring system for each patient group.

Cytomegalovirus DNA cut-off values can be used to facilitate early diagnosis and treatment, thus improving patient management and outcomes. In this study, a plasma CMV DNA of 272 copies/mL was defined as an optimal cut-off for predicting CMV-GID prior to endoscopic and histopathological examinations. Endoscopic examination should not be underestimated for differential diagnosis. The presence of ulcers, exudating ulcers, ulcers with irregular margins, large mucosal defects, macroscopic hemorrhage, and pancolitis is the main endoscopic finding suggesting CMV-GID. The diagnosis of CMV-GID can be achieved using the proposed scoring system, which takes into account the age, systemic steroid use, viral load, and endoscopic examination findings. Particularly in cases where endoscopic examination and a definitive histopathological diagnosis are not possible or where early diagnosis is crucial to preventing complications or fatal outcomes, the scoring system can help clinicians make a preliminary diagnosis of CMV-GID if the patient’s CMV DNA levels are above the cut-off values predictive of the disease. However, if the patient’s CMV DNA level is below the cut-off values, the disease cannot be completely excluded. In such a case, CMV-GID can be highlighted in the clinician’s preliminary diagnosis by calculating the score using the recommended scoring system after endoscopic examination in appropriate patients while awaiting the pathology report. It should be noted that the definitive diagnosis of CMV-GID can still be made by histopathological examination, and both the CMV DNA cut-off values and the scoring system can be regarded as adjunctive diagnostic tools.

## Supplementary Materials

Supplementary Material

## Figures and Tables

**Table 1.** Demographic and Clinical Characteristics of the Study Patients with Respect to Cytomegalovirus Gastrointestinal Disease Status*

		CMV-GID n = 125	Non-CMV-GID n = 177	Total n	*P*
Age, years		58 (29)	43 (27)	–	**<.001**
Age, n (%)	18-30	16 (32)	34 (68)	50	**<.001**
	31-50	31 (29.2)	75 (70.8)	106	
	51-65	39 (49.4)	40 (50.6)	79	
	65 +	39 (58.2)	28 (41.8)	67	
Sex, n (%)	Male	78 (44.6)	97 (55.4)	175	.188
	Female	47 (37)	80 (63)	127	
Underlying disease, n (%)	IBD	40 (46)	47 (54)	87	.252
	SOT	9 (39.1)	14 (60.9)	23	
	HSCT	11 (25)	33 (75)	44	
	Solid organ malignancy	21 (53.9)	18 (46.1)	39	
	Hematologic malignancy	6 (35.3)	11 (64.7)	17	
	HIV/AIDS	6 (40)	9 (60)	15	
	Rheumatological disease/autoimmunity/primary immunodeficiency	15 (37.5)	25 (62.5)	40	
	Multimorbidity**/immunocompetent host	17 (46)	20 (54)	37	
Type of IBD, n (%)	Ulcerative colitis	33 (47.1)	37 (52.9)	70	.658
	Crohn’s disease	7 (41.2)	10 (58.8)	17	
Systemic steroid use, n (%)		70 (53)	62 (47)	132	**.001**
Azathioprine use, n (%)		31 (54.4)	26 (45.6)	57	**.027**
Mycophenolate mofetil use, n (%)		10 (35.7)	18 (64.3)	28	.522
Cyclosporine use, n (%)		9 (32.1)	19 (67.9)	28	.297
Tacrolimus use, n (%)		7 (29.2)	17 (70.8)	24	.205
Use of more than 2 immunosuppressive drugs n (%)		70 (48.6)	74 (51.4)	144	**.015**
Type of HSCT, n (%)	Allogeneic	10 (25)	30 (75)	40	.999
	Autologous	1 (25)	3 (75)	4	

IBD, Inflammatory bowel disease; SOT, Solid organ transplantation; SHSCT, aematopoetic stem cell

*Median (interquartile range) for continuous variables; frequency (percentage) for categorical variables.

**Two or more comorbidities other than immunosuppressive status.

Bold are all statistically significant values.

**Table 2.** Endoscopic Examination Findings of the Study Patients with Respect to Cytomegalovirus Gastrointestinal Disease Status

	CMV-GID n = 125	Non-CMV-GID n = 177	Total n	*P*
Hyperemia/edema, n (%)	82 (40.8)	119 (59.2)	201	.767
Erosion, n (%)	35 (50.72)	34 (49.28)	69	.073
Granular appearance, n (%)	16 (55.17)	13 (44.83)	29	.113
Ulcer, n (%)	99 (79.2)	103 (58.2)	202	**<.001**
Exudative ulcer, n (%)	41 (60.29)	27 (39.71)	68	**<.001**
Aphthous ulcer, n (%)	6 (40)	9 (60)	15	.911
Ulcer with irregular margin, n (%)	25 (75.76)	8 (24.24)	33	**<.001**
Large mucosal defect, n (%)	46 (90.2)	5 (9.8)	51	**<.001**
Pseudopolyps, n (%)	12 (50)	12 (50)	24	.372
Tumor/polypoid formation n, (%)	9 (39.13)	14 (60.87)	23	.819
Fragile mucosa, n (%)	21 (40.38)	31 (59.62)	52	.871
Disappearance of submucosal vasculature, n (%)	21 (45.65)	25 (54.35)	46	.524
Bleeding, n (%)	21 (56.76)	16 (43.24)	37	**.043**
Pancolitis, n (%)	19 (67.86)	9 (32.14)	28	**.003**
Perforation, n (%)	9 (56.25)	7 (43.75)	16	.215

Bold are all statistically significant values.

**Table 3.** Cytomegalovirus DNA Cut-Off Values and Performance Measures to Predict Cytomegalovirus Gastrointestinal Disease

Cut-off Value		Area Under Curve	*P*	Power	Sensitivity	Specificity	Positive Predictive Value	Negative Predictive Value
Youden Index	272 cp/mL	0.787	<.001	1.000	0.608	0.876	0.776	0.760
Maximum Sensitivity	9 cp/mL				0.792	0.621	0.596	0.809
Maximum Specificity	611 cp/mL				0.528	0.927	0.835	0.735

**Table 4.** Odds Ratios in the Univariate Analyses of Factors with Possible Associations with Cytomegalovirus Gastrointestinal Disease

	Odds Ratio (OR)	95% CI
65+ years	2.22	1.28-3.83
Systemic steroid use	2.36	1.47-3.74
Erosion	1.63	0.95-2.80
Ulcer	2.73	1.61-4.62
Pancolitis	3.34	1.46-7.67
Granular appearance	1.85	0.85-4.00

**Table 5.** Proposed Scoring System to Predict Cytomegalovirus Gastrointestinal Disease

		*β*	SE*(β)*	*P*	Exp(*β*)	95% CI		**Score**
						Lower Limit	Upper Limit	
Constant	−2.884	0.372	<0.001	.056
		
Age	Age of diagnosis (+65 years)	0.827	0.349	.018	2.285	1.153	4.531	**8**
Drug use	Systemic steroid use	0.511	0.300	.088	1.668	0.926	3.002	**5**
Endoscopic examination findings	Erosion	0.863	0.324	.008	2.369	1.256	4.472	**9**
	Ulcers	1.071	0.533	.045	2.917	1.026	8.299	**11**
	Pancolitis	0.602	0.348	.084	1.826	0.922	3.616	**6**
	Granular appearance	0.956	0.480	.046	2.602	1.015	6.673	**10**
Viral load monitoring	Increase of 1log_10 _copies/mL	0.723	0.100	<.001	2.060	1.694	2.505	**7**

Bold are all statistically significant values.

**Table 6.** Cut-Off Values of the Proposed Score to Predict Cytomegalovirus Gastrointestinal Disease

	Cut-Off	C Index	*P*	CI		Sensitivity	Specificity	Positive Predictive Value	Negative Predictive Value
				Lower Limit	Upper Limit				
Youden Index	28.1	0.825	<.0001	0.776	0.871	0.704	0.870	0.793	0.806
Maximum sensitivity	17					0.864	0.531	0.565	0.847
Maximum specificity	37.4					0.520	0.960	0.903	0.739

## Data Availability

The data that support the findings of this study are available on request from the corresponding author.
