# Supplementary material for: Is It Possible to Determine Plasma Cytomegalovirus DNA Cut-off and Develop a Scoring System to Predict Cytomegalovirus Gastrointestinal Disease?
Source: Turk J Gastroenterol. 2025 Oct 6;36(12):798–806. doi: 10.5152/tjg.2025.24507 (PMC12684270; doi:10.5152/tjg.2025.24507)
Supplement: Supplementary Material [file supplementary_material.pdf]

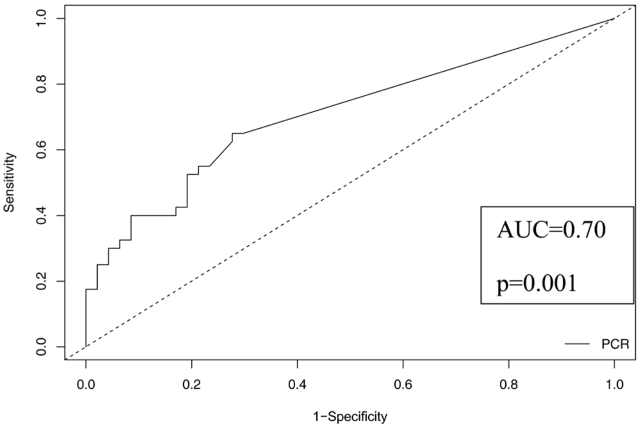

Supplementary Figure 1. ROC curve of CMV DNA in plasma for the patients with IBD

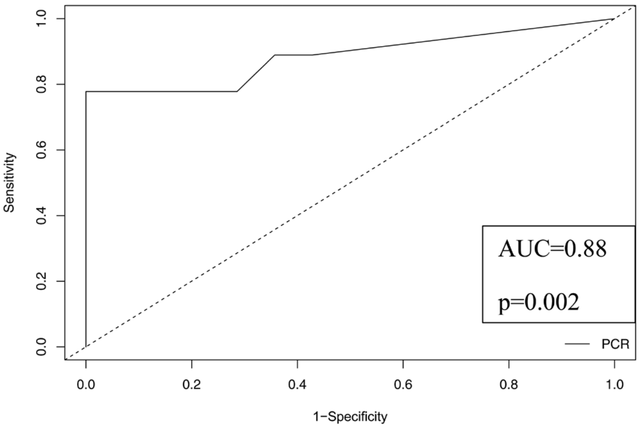

Supplementary Figure 2. ROC curve of CMV DNA in plasma for the SOT recipients.

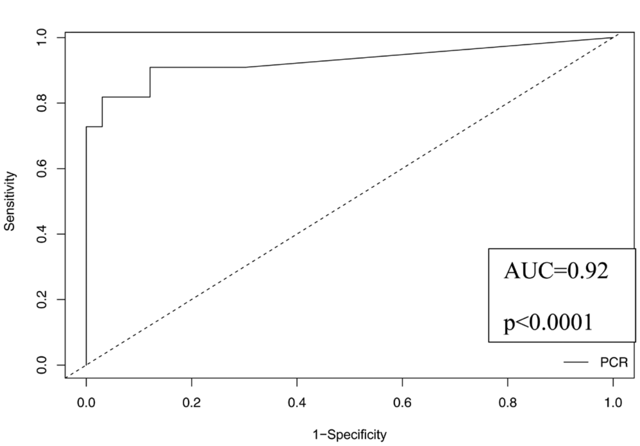

Supplementary Figure 3. ROC curve of CMV DNA in plasma for the HSCT recipients.

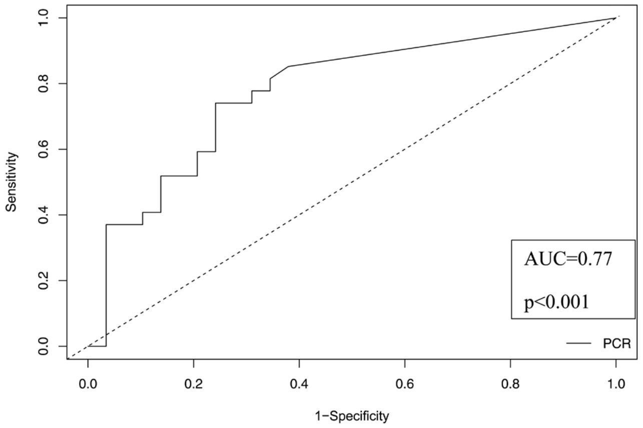

Supplementary Figure 4. ROC curve of CMV DNA in plasma for the patients with solid organ and hematologic malignancies.

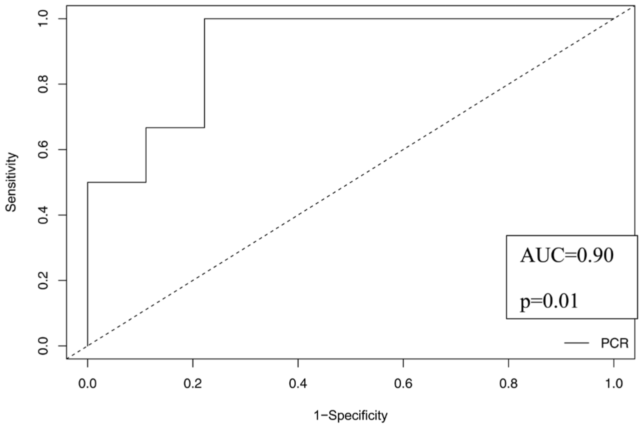

Supplementary Figure 5. ROC curve of CMV DNA in plasma for the HIV and AIDS patients.

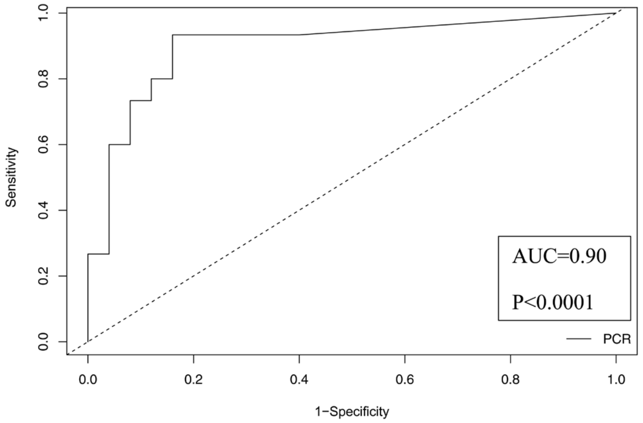

Supplementary Figure 6. ROC curve of CMV DNA in plasma for the patients with rheumatologic and autoimmune diseases and primary immunodeficiencies.

Supplementary Table 1. Distribution of gastrointestinal complaints of the study patients with respect to CMV-GID status

	CMV-GID n=125	Non-CMV-GID n=177	Total n	*p*
Fever, n (%)	34 (42.50)	46 (57.50)	80	0.814
Dyspepsia, n (%)	17 (62.96)	10 (37.04)	27	**0.017**
Regurgitation, n (%)	6 (54.55)	5 (45.45)	11	0.372
Dysphagia, n (%)	13 (43.33)	17 (56.67)	30	0.820
Odynophagia, n (%)	11 (45.83)	13 (54.17)	24	0.645
Abdominal pain, n (%)	54 (42.86)	72 (57.14)	126	0.662
Nausea and/or vomiting, n (%)	36 (36.73)	62 (63.27)	98	0,.55
Diarrhea, n (%)	65 (36.72)	112 (63.28)	177	0.050
Constipation, n (%)	8 (61.54)	5 (38.46)	13	0.132
Gastrointestinal bleeding, n (%)	62 (46.62)	71 (53.38)	133	0.102
Weight loss, n (%)	26 (44.07)	33 (55.93)	59	0.642

Supplementary Table 2. Site of gastrointestinal involvement of the study patients with respect to CMV-GID status

	CMV-GID n=125	Non-CMV-GID n=177	Total n	*p*
Esophagus, n (%)	18 (38.3)	29 (61.7)	47	0.639
Stomach, n (%)	21 (44.68)	26 (55.32)	47	0.618
Duodenum, n (%)	5 (25)	15 (75)	20	0.124
Upper GI tract, n (%)	38 (40.86)	55 (59.14)	93	0.901
Ileum, n (%)	9 (23.68)	29 (76.32)	38	**0.018**
Caecum, n (%)	7 (35)	13 (65)	20	0.548
Colon, n (%)	71 (40.8)	103 (59.2)	174	0.809
Rectum, n (%)	30 (38.46)	48 (61.54)	78	0.542
Lower GI tract, n (%)	96 (41.56)	135 (58.44)	231	0.915

Supplementary Table 3. Laboratory findings of the study patients at the time of diagnosis with respect to CMV-GID status

	CMV-GID*	CMV-GID n	Non-CMV-GID *	Non-CMV-GID n	*p*
Hemoglobin (g/dL)	10.1 (3.1)	125	10.9 (3.6)	177	0.288
Leukocyte ($\times10^3/\mu L$)	6.7 (4.4)	125	6.9 (5.6)	177	0.439
Lymphocyte ($\times10^3/\mu L$)	1.1 (1.3)	125	1.2 (1.1)	177	0.078
Neutrophil ($\times10^3/\mu L$)	4.6 (4.2)	125	4.7 (4.6)	177	0.807
Eosinophil ($\times10^3/\mu L$)	0.03 (0.12)	125	0.1 (0.1)	177	0.439
Platelets ($\times10^3/\mu L$)	227 (193)	125	220 (191)	177	0.951
CRP (mg/dL)	3.32 (7.52)	121	3.49 (8.09)	169	0.486
Erythrocyte sedimentation rate (mm/h)	26 (32.0)	121	24 (32.5)	168	0.760
Alanine aminotransferase (U/L)	16 (19)	125	16 (20)	177	0.935
Aspartate aminotransferase (U/L)	22 (12.5)	124	20 (16.0)	177	0.434
Alkaline phosphatase (U/L)	91.5 (76)	124	86.0 (67)	175	0.216
Gamma glutamate transferase (U/L)	39 (87)	123	36 (71)	175	0.373
Albumin (g/dL)	2.89 (1.19)	125	3.11 (1.15)	171	0.134
Total bilirubin (mg/dL)	0.58 (0.47)	121	0.59 (0.43)	172	0.369
Creatinine (mg/dL)	0.73 (0.42)	125	0.75 (0.57)	174	0.295
CD4+ T lymphocyte (cell/mm^3)	29 (87)	6	134 (112)	9	0.077
CD4+ T lymphocyte count <50 cell/mm^3	3 (42.9)	6	4 (57.1)	9	0.999

* Median (interquartile range) for continuous variables; frequency (percentage) for categorical variables.

Supplementary Table 4. CMV DNA cut-off values and performance measures to predict CMV-GID for each subgroup of the study patients

Diagnostic category	Cut-off (Youden)	AUC	*p*	Power	Sensitivity	Specificity	Positive Predictive Value	Negative Predictive Value
1	9	0.706	0.001	0.933	0.650	0.723	0.667	0.708
2	298	0.885	0.002	0.955	0.778	1.000	1.000	0.875
3	290	0.927	<0.001	1.000	0.909	0.879	0.714	0.967
3	875	0.927	<0.001	1.000	0.818	0.970	0.900	0.941
4 and 5	124	0.777	<0.00001	0.972	0.741	0.759	0.741	0.759
6	1447	0.907	0.010	0.881	1.000	0.778	0.750	1.000
7	86	0.900	<0.001	1.000	0.933	0.840	0.778	0.955
8*	-	0.710	0.029	0.622	-	-	-	-

*The optimal cut-off value for the immunocompetent hosts (diagnosis code 8) was not presented since the post-hoc power analysis was below 80%.

Annex-1: Case Annex-1: Case Report Form

The patient's anonymous code

Diagnosis of CMV-GID (yes/no)

The date of receipt of the biopsy specimen (dd.mm.yyyy)

Date of CMV DNA in plasma (dd.mm.yyyy)

Plasma CMV DNA (copies/mL) – most recent to the biopsy date

Elapsed time between tissue sample collection and plasma CMV DNA (days)

Age at diagnosis (years)

Sex (female/male)

Underlying/concomitant diseases

Category of the primary disease considered among major risks for CMV-GID	1: Inflammatory bowel disease (IBD)<br>2: Solid organ transplant recipient (SOT)<br>3: Hematopoietic stem cell transplant recipient (HSCT)<br>4: Solid organ malignancy<br>5: Hematological malignancy without hematopoietic stem cell transplantation<br>6: HIV&AIDS<br>7: Autoimmune and rheumatological diseases/primary immunodeficiencies<br>8: Other comorbidities / immunocompetent persons

Type of IBD, if selected (UC/CH)

Refractoriness to steroid therapy if IBD selected (yes/no)

HSCT (yes/no)

Type of HSCT if performed (allo/auto)

Graft versus host disease (yes/no)

GI GVHD (yes/no) if GVHD is present

Solid organ transplant (yes/no)

Type of SOT if performed (kidney/liver/other)

Rejection if SOT performed (yes/no)

CD4+ T lymphocyte count (cell/mm^3) for HIV/AIDS patients

Steroid use (yes/no)

Azathioprine use (yes/no)

Mycophenolate mofetil use (yes/no)

Calcineurin inhibitor use (e.g., cyclosporine, tacrolimus) (yes/no)

Use of more than two immunosuppressives (yes/no)

Valacyclovir use (yes/no)

Complaints	Fever<br>Dyspepsia<br>Regurgitation<br>Dysphagia<br>Odynophagia<br>Abdominal pain<br>Nausea and/or vomiting<br>Diarrhea<br>Constipation<br>GI bleeding<br>Weight loss

Other complaints if any

Type of endoscopic examination (Upper/Lower/Upper&Lower)

Endoscopic examination note	Hyperemia and edema (yes/no)
	Erosion (yes/no)
	Granular appearance (yes/no)
	Ulcer (yes/no)
	Exuding ulcer (yes/no)
	Aphthous ulcer (yes/no)
	Ulcer with irregular margin (yes/no)
	Large mucosal defect (yes/no)
	Pseudotumor (yes/no)
	Tumor or polypoid formation (yes/no)
	Fragile mucosa (yes/no)
	Deletion of submucosal blood vessels (yes/no)
	Macroscopic bleeding (yes/no)
	Pancolitis (yes/no)
	Perforation (yes/no)
	Other
Report of histopathological and immunohistochemical findings	Inclusion body (yes/no)
	Cytomegaly (yes/no)
	Giant cell (yes/no)
	Other
Immunohistochemical staining result (Positive/Negative)	
GI involvement site	Upper GI involvement (yes/no)
	Oral involvement (yes/no)
	Esophageal involvement (yes/no)
	Gastric involvement (yes/no)
	Duodenal involvement (yes/no)
	Lower GI involvement (yes/no)
	Ileal involvement (yes/no)
	Cecal involvement (yes/no)
	Appendiceal involvement (yes/no)
	Colonic involvement ((yes/no)
	Rectal involvement (yes/no)
	Anal involvement (yes/no)
CMV IgM result (Positive/Negative)	
CMV IgG result (Positive/Negative)	
Hemoglobin level (g/dL)	
Leukocyte count ($\times 10^3/\mu L$)	
Lymphocyte count ($\times 10^3/\mu L$)	
Neutrophil count ($\times 10^3/\mu L$)	
Eosinophil count ($\times 10^3/\mu L$)	
Platelet count ($\times 10^3/\mu L$)	
CRP level (mg/dL)	
Erythrocyte sedimentation rate (mm/hour)	
Alanine aminotransferase level (U/L)	
Aspartate aminotransferase level (U/L)	
Alkaline phosphatase level (U/L)	
Gamma-glutamyl transferase level (U/L)	
Albumin level (g/dL)	
Total bilirubin level (mg/dL)	
Creatinine level (mg/dL)	
Tissue CMV DNA, if present (copies/mL)	
Plasma CMV DNA monitoring values (copies/mL)	
Peak plasma CMV DNA (copies/ml)	
Non-GI CMV disease involvement (yes/no)	
Involvement site if non-GI involvement	
Antiviral treatment (yes/no)	
Name of antiviral drug (ganciclovir/valganciclovir/foscarnet/other)	
Initial date of antiviral treatment (dd.mm.yyyy)	
The total duration of antiviral treatment (days)	
Follow-up endoscopic examination (yes/no)	
Report of follow-up endoscopic examination if performed	
Follow-up endoscopic biopsy (yes/no)	
Report of follow-up endoscopic biopsy if performed	
Improvement in follow-up endoscopic examination findings (yes/no)	
Persistence of CMV positivity in follow-up endoscopic biopsy (yes/no)	
Improvement in the patient complaints (yes/no)	
Requirement for surgical intervention during an attack (yes/no)	
Hospitalization period of the patient during the attack (days)	
Mortality during the attack (yes/no)	
Non-CMV etiology in the absence of CMV-GID	
